# Diversity of the microbiota communities found in the various regions of the intestinal tract in healthy individuals and inflammatory bowel diseases

**DOI:** 10.3389/fimmu.2023.1242242

**Published:** 2023-11-02

**Authors:** Samuel Adefisoye Lawal, Athalia Voisin, Hana Olof, Michael Bording-Jorgensen, Heather Armstrong

**Affiliations:** ^1^ Department of Medical Microbiology and Infectious Diseases, University of Manitoba, Winnipeg, MB, Canada; ^2^ Manitoba Centre for Proteomics and Systems Biology, University of Manitoba, Winnipeg, MB, Canada; ^3^ IBD Clinical and Research Centre, University of Manitoba, Winnipeg, MB, Canada; ^4^ Department of Immunology, University of Manitoba, Winnipeg, MB, Canada; ^5^ Department of Pediatrics, University of Alberta, Edmonton, AB, Canada; ^6^ Department of Internal Medicine, University of Manitoba, Winnipeg, MB, Canada

**Keywords:** IBD, microbiome, dysbiosis, bacteriome, mycobiome, virome

## Abstract

The severe and chronic inflammatory bowel diseases (IBD), Crohn disease and ulcerative colitis, are characterized by persistent inflammation and gut damage. There is an increasing recognition that the gut microbiota plays a pivotal role in IBD development and progression. However, studies of the complete microbiota composition (bacteria, fungi, viruses) from precise locations within the gut remain limited. In particular, studies have focused primarily on the bacteriome, with available methods limiting evaluation of the mycobiome (fungi) and virome (virus). Furthermore, while the different segments of the small and large intestine display different functions (*e.g.*, digestion, absorption, fermentation) and varying microenvironment features (*e.g.*, pH, metabolites), little is known about the biogeography of the microbiota in different segments of the intestinal tract or how this differs in IBD. Here, we highlight evidence of the differing microbiota communities of the intestinal sub-organs in healthy and IBD, along with method summaries to improve future studies.

## Introduction

Dysbiosis (altered abundance and diversity of microbiota; bacteria, fungi, and viruses) is a known hallmark of the inflammatory bowel diseases (IBD), Crohn disease (CD) and ulcerative colitis (UC) (1). However, the precise definition of a healthy and diseased microbiome remains poorly defined (2). It is well recognized that this is in part due to the significant inter- and intra-individual heterogeneity of the microbiome (microbiota, microenvironment, interactions with the host), along with limitations in sampling and processing techniques (3, 4). Yet, one factor that remains largely overlooked is the significant diversity of the microbiome in the various subsections of the intestinal tract, with many studies describing sample collection from only the “small intestine” or “large intestine”. Here we summarize what is currently known about the variations in the composition of the microbiota communities identified at specific sites along the gastrointestinal (GI) tract in healthy individuals and patients living with IBD.

## The functions of the subsections of the small and large bowels

**Figure 1:**
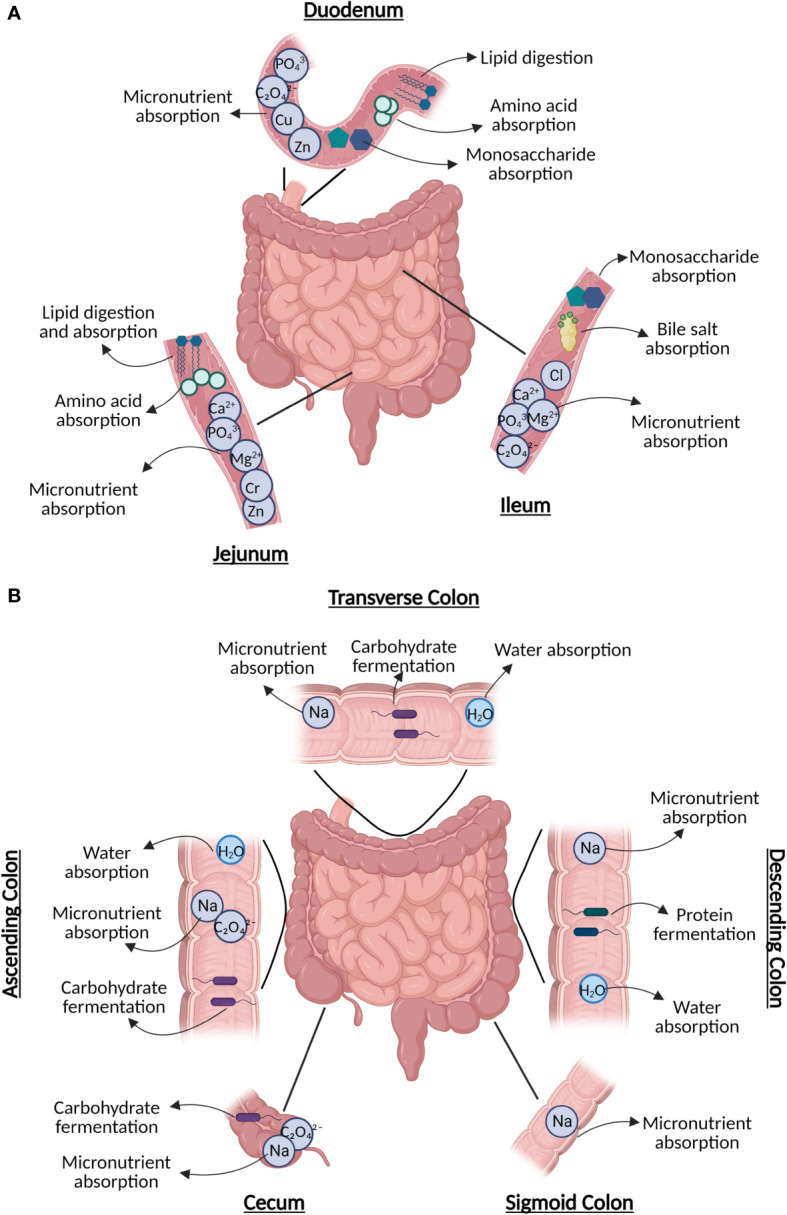
The diverse roles of subsections of the **(A)** small intestine, including digestion (duodenum), nutrient absorption (duodenum/jejunum), lipid digestion (jejunum), and sugar absorption (ileum); and **(B)** large intestine, including water absorption (every section), carbohydrate fermentation, and remaining nutrient absorption. Figure created in BioRender.

Food substrates, host cells, and luminal and mucosal gut microbiota come into close contact throughout the intestinal tract, generating a microenvironment rich in microbe–microbe and host–microbe interactions, which are closely linked to health and disease outcomes (5, 6). It is important to recognize the segments of the intestinal tract include the duodenum, jejunum, and ileum, which make up the small intestine; while the cecum, ascending colon (ASC), transverse colon, descending colon, sigmoid colon, and rectum make up the large intestine. These organs serve diverse roles from digestion, to absorption of nutrients and water, to microbial fermentation of proteins and fibers (**Figure 1**) (7, 8). Previous review articles have highlighted the in-depth physiology of these organs (7, 8) however, here we will briefly highlight their roles to better support discussion of the diverse microbiota within the segments of the small and large intestines.

### Small intestine

The duodenum is the first and shortest portion of the small intestine, which plays a crucial role in the digestion of food contents exiting the stomach with assistance from pancreatic secretions containing digestive enzymes (**Figure 1A**) (9). Both the duodenum and jejunum (the mid-segment of the small intestine) are responsible for the bulk of nutrient absorption and assimilation (10). Further, the jejunum is also responsible for the absorption and digestion of most dietary lipids (11). The most distal segment of the small intestine, known as the ileum, is involved in the absorption of bile acids and simple sugars (6, 12). The ileum also contains the collection of lymphoid follicles located in the mucus membrane known as Peyer’s patches, which are master immune regulators of the intestine where interactions occur between antigens and microbiota with immune cells. These interactions are mediated by both nucleotide-binding oligomerization domain two (NOD2), a pattern recognition cytosolic protein highly expressed in the ileal Paneth cells with its loss of function linked to CD, and other pathogen recognition receptors that can also be altered in IBD, resulting in abnormal responses targeting commensal microbiota (13–15).

### Large intestine

The ileocecal valve, which joins the small and large intestines, shields the opening of the ileum into the cecum (8). While the bulk of digestion and absorption of food occurs in the small intestine, the large intestine aids in final water absorption and waste removal (**Figure 1B**) (8). The proximal parts of the colon (cecum, ASC, and transverse colon) are responsible for carbohydrate fermentation by microbiota, producing short-chain fatty acids (SCFA)s (16). Protein fermentation producing branched-chain fatty acids typically occurs in the distal descending and sigmoid segments of the colon (16). The colon is also responsible for obtaining key vitamins, such as cobalamin (B_12_) found in animal products, yeast, and algae, along with minerals such as calcium (17). Further, the ASC is responsible for the absorption of sodium (Na^+^) via electroneutral sodium-chloride transport, and the descending colon has been reported to be associated with amiloride-insensitive Na^+^ absorption (17).

## Microbiota profiles of the small intestine

**Figure 2:**
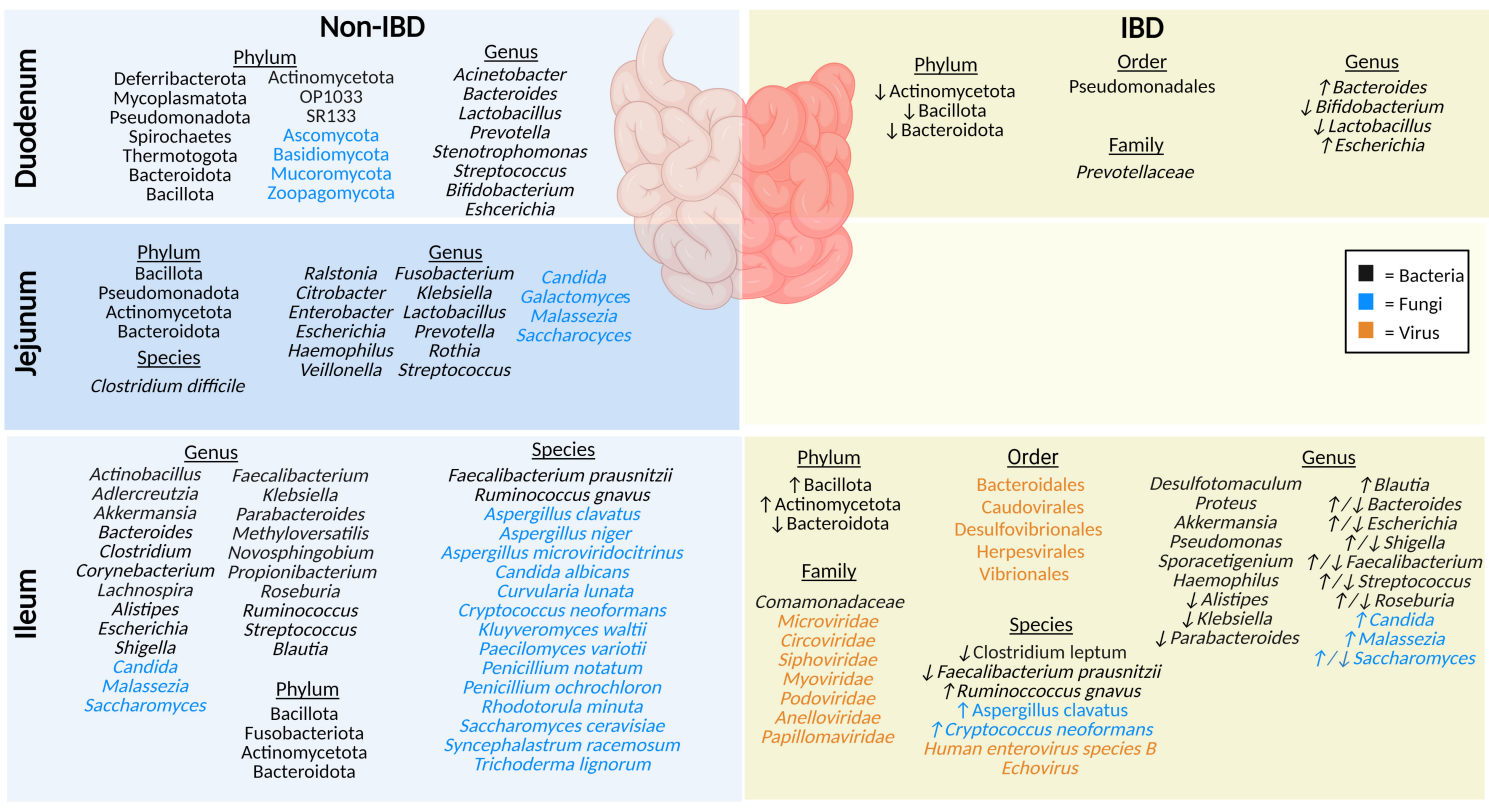
The microbiota populations previously identified within the different sections of the small intestine in non-IBD (left) and IBD (right) patients. Black text (bacteria), blue text (fungi), orange text (viruses). Figure created in BioRender.

Due to sampling challenges, including the inability to easily access the small intestine via endoscopy (proximal duodenum) or colonoscopy (terminal ileum), limited research on the microbiota of the small intestine has been performed (18). Hence, there is often a reliance on animal models which do not completely reflect human intestinal microbiota (19, 20). The microenvironment of the small intestine is less favorable for microbial growth than the colon due to the lower pH, increased concentration of oxygen, and antimicrobial peptides produced by host cells of the epithelial lining of the small intestine such as α- defensins, C-type lectins interfacing as a shield against pathogenic microbes (21, 22). As such, most microbes in the small intestine are fast-growing, facultative anaerobes (21). Generally, microbial abundance increases significantly after exiting the duodenum (10^1^-10^3^) CFU/ml) and continuing to the jejunum (10^4^-10^7^ CFU/ml) and ileum (10^3^–10^8^ CFU/ml) (23). Below we discuss the key microbial species identified in healthy sections of the small intestine and the changes reflected in IBD (**Figure 2**).

### Duodenum

The duodenum is located between the acid-secreting stomach and the nutrient-absorbing jejunum, therefore participating in continued digestion and nutrient absorption, displaying a lower overall abundance of microbes compared to the rest of the intestinal tract, yet greater diversity (by phyla) than the rectum (24). The bacteriome (16S rRNA) of healthy adults profiled in biopsy tissues shows the duodenum is chiefly dominated by phyla Pseudomonadota (formerly Proteobacteria), Bacteroidota (formerly Bacteroidetes), Actinomycetota (formerly Actinobacteria), and Bacillota (formerly Firmicutes), along with the genera *Acinetobacter, Bacteroides, Prevotella*, *Bifidobacterium, Escherichia*, and *Lactobacillus* (24–26). Luminal (mucus) duodenum samples predominantly house genera *Stenotrophomonas* and *Streptococcus* (24). Interestingly, one small study (9 participants) using Chinese healthy volunteers identified several rare bacterial phyla (OP10, SR1, Mycoplasmatota [formerly Tenericutes], Thermotogota [formerly Thermotogae], Deferribacterota, and Spirochaetes), and noted that the microbial samples they collected from biopsies were more conserved than luminal mucosal samples (24). Some of the reasons underlying this variable microbial profile (biopsies vs mucosal) may include the rapid transit time of luminal contents, low pH, and high concentrations of bile acids, digestive enzymes, host-defense peptides (HDPs), and immunoglobulins (21, 27). This microenvironment reduces microbial colonization, while certain phyla of fungi, such as Ascomycota, Basidiomycota, Mucoromycota and Zoopagomycota (formerly Zygomycota), can thrive in these low pH conditions (27, 28). There are currently no studies on the virome (eukaryotic viruses or bacteriophages) in the duodenum of healthy individuals.

In contrast, IBD patients have a lower abundance of mucosal duodenal bacteria (25). Beneficial genera of bacteria *Bifidobacterium* and *Lactobacillus* are notably decreased in IBD, whereas the populations of *Bacteroides* and *Escherichia* genera are increased (25). Furthermore, F. Sjöberg et al. performed a novel pilot study where luminal fluids were sampled from treatment-naïve children who were suspected of having IBD, highlighting a low richness and a reduced prevalence of Actinomycetota (formerly Actinobacteria), Bacteroidota (formerly Bacteroidetes), and Bacillota (formerly Firmicutes) phyla (26). Limited studies highlight key differences in the duodenal microbiota compared to other segments of the intestinal tract, and in IBD compared to non-IBD. The duodenal mycobiome and virome (eukaryotic viruses or bacteriophages) in individuals with IBD has not been defined.

### Jejunum

The jejunum is a structurally and functionally distinct region of the small intestine, involved in nutrient absorption such as magnesium and phosphate, along with absorption and digestion of most dietary lipids (10, 11). In healthy individuals, the microbiota plays a crucial role in lactate production, which is an important energy source for stem cells in the small intestine (11). Unfortunately, sampling difficulties account for one of the reasons why the jejunal microbiota is understudied. The jejunal microbiota of healthy humans includes a high abundance of members of phyla Bacillota (formerly Firmicutes), Pseudomonadota (formerly Proteobacteria), Actinomycetota (formerly Actinobacteria), and Bacteroidota (formerly Bacteroidetes) (23). To a lesser extent, other detected genera include *Enterobacter, Escherichia, Lactobacillus, Streptococcus, Klebsiella, Veillonella, Fusobacterium, Rothia, Prevotella, Ralstonia*, *Haemophilus* and *Citrobacter*, and the species *Clostridium difficile* (29–31). In addition, a recent review article highlighted fungal genera including *Malassezia, Candida, Saccharomyces*, and *Galactomyces* in the jejunum of healthy individuals (32). While the jejunal microbiota has not been defined in IBD patients, damage and inflammation during active disease result in the reduction of the epithelial barrier of the jejunum, allowing entry of microbial lipopolysaccharides, demonstrating links between the microbiota and IBD (1, 11). In addition, the jejunal mycobiome in IBD patients and the virome (eukaryotic viruses or bacteriophages) in both healthy and IBD individuals have not been described thus far.

### Ileum

Although any part of the GI tract may be affected by CD, the terminal ileum is the most commonly affected area in CD pathogenesis (33). In a healthy individual, the ileum plays a significant role in the absorption of simple sugars and bile acids, which is significantly altered in ileal CD and may have a significant impact on luminal bacteria and fungi in particular (12, 34). Phyla identified using 16S sequencing of ileal mucosa in healthy adults include Actinomycetota (formerly Actinobacteria), Bacteroidota (formerly Bacteroidetes), Bacillota (formerly Firmicutes), and Fusobacteriota (35). The healthy ileal bacteriome is dominated by a high abundance of the genera *Clostridioides, Streptococcus, Bacteroides*, and *Corynebacterium* (35, 36). Other bacteria identified in the ileal mucosa include the genera *Alistipes*, *Blautia*, *Escherichia, Shigella, Faecalibacterium, Klebsiella, Parabacteroides, Actinobacillus*, *Novosphingobium*, *Methyloversatilis*, *Akkermansia*, *Propionibacterium*, *Ruminococcus*, *Aldercreutzia*, *Lachnospira*, and *Roseburia;* in particular the species *Ruminococcus gnavus* and *Faecalibacterium prausnitzii* (35, 37). The ileal lumen of healthy individuals also houses fungi from the genera *Saccharomyces, Malassezia*, and *Candida*, along with the species *Saccharomyces cerevisiae, Aspergillus clavatus, Aspergillus niger, Candida albicans, Curvularia lunata, Penicillium notatum, Penicillium ochrochloron, Kluyveromyces waltii*, and a smaller percentage of species including *Paecilomyces variotii, Aspergillus microviridocitrinus, Rhodotorula minuta, Trichoderma lignorum, Syncephalastrum racemosum* and *Cryptococcus neoformans* (32, 38–41). The ileal virome was examined in healthy control stool but has not been precisely examined in the ileum using appropriate sampling techniques to date (42).

The microbiota composition of the ileum is notably different in individuals with IBD. This includes an increase in Actinomycetota (formerly Actinobacteria) and Bacillota (formerly Firmicutes), and a reduction in Bacteroidota (formerly Bacteroidetes) in ileal mucosa (35). At the family level, ileal bacteria *Comamonadaceae* have been described, and the IBD mucosa include the genera *Proteus* and *Desulfotomaculum* (37). Many other genera have been specifically identified in IBD only; for example, *Akkermansia* were only found in IBD ileal mucosal samples and the genera *Pseudomonas, Haemophilus*, and *Sporacetigenium* were only found in UC ileal mucosal samples (35). The genera *Alistipes, Klebsiella*, and *Parabacteroides* were decreased in IBD patient ileal mucosal samples, and *Blautia* and *Roseburia* were increased (35). In contrast, another study found a reduction in IBD mucosal genera *Roseburia* (39). Meanwhile, the *Bacteroides* genera was decreased in UC mucosal samples but increased in CD, and *Shigella, Escherichia, Faecalibacterium*, and *Streptococcus* were decreased in CD mucosal samples but increased in UC samples (35, 37). At the species level, in ileal IBD samples there was elevated mucosal species *Ruminococcus gnavus* along with reduced mucosal species *Clostridium leptum*, and reduced luminal *Faecalibacterium prausnitzii* (39, 41). The mycobiome is significantly different between CD and non-IBD ileal biopsy samples; a high abundance of the genus *Saccharomyces* and the species *Aspergillus clavatus*, and *Cryptococcus neoformans* was identified (39). In contrast, another study found a decrease in the abundance of *Saccharomyces* and an increase of *Malassezia* and *Candida* in CD patient biopsies compared to healthy controls (40). Comparing virome results of CD biopsies against that of healthy control stool samples, demonstrated that ileal biopsies from active CD patients had a high abundance of bacteriophages and eukaryotic viruses from the order Caudovirales, Bacteroidales, Herpesvirales, Vibrionales, and Desulfovibrionales, and the families *Microviridae*, *Circoviridae*, *Anelloviridae*, *Papillomaviridae*, among other unidentified viruses (42). A study examined bacteriophages present in biopsies and gut wash samples from pediatric CD patients and identified bacteriophages from the Caudovirales order (*Myoviridae, Siphoviridae*, *Podoviridae*) in the ileum (43). At the species level, *Human Enterovirus* species B and *Echovirus* were also identified in ileal biopsies collected from advanced ileocecal CD patients (44). Sampling difficulties have led many studies to compare and contrast the small intestine as a whole between healthy and IBD patients, when the sample likely represents the terminal ileum collected during colonoscopy (5, 45). Furthermore, differences in sampling techniques and sites (*e.g.*, biopsies, gut washes, gut brushings) have resulted in conflicting results across studies, particularly when compared to stool from healthy controls (42, 46). While these studies are excellent examples of the variation that occurs between the intestinal sub-organs, and in IBD patients, improved sampling techniques, highlighted by Tang et al. (47), are sure to broaden our understanding of the precise role of the duodenal, jejunal, and ileal microbiota in IBD in future.

## Microbiota profiles of the large intestine

**Figure 3:**
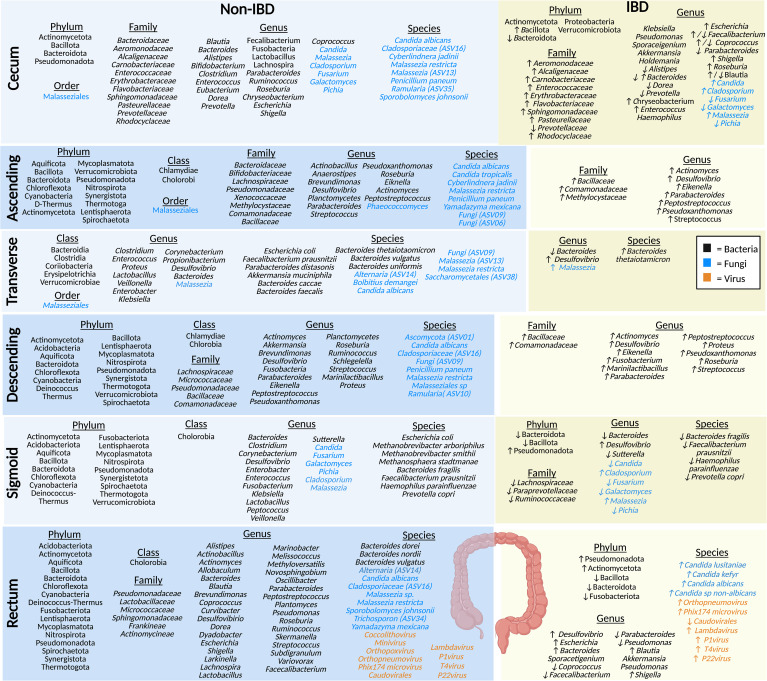
The microbiota populations previously identified within the different sections of the large intestine in non-IBD (left) and IBD (right) patients. Black text (bacteria), blue text (fungi), orange text (viruses). Figure created in BioRender.

Regional differences are particularly noticeable when comparing the segments of the colon because microbial diversity progressively increases from the proximal to the distal colon (23, 48). The colon is a more conducive habitat for microbiota growth compared to the small intestine because it has a longer transit time and higher pH, a lower cell turnover, a lower redox potential, and fewer antimicrobials (21, 49). In this microenvironment, many bacteria in the colon are fermentative, polysaccharide-degrading anaerobes (21). Interestingly, the colonic mucosal mycobiome displays an overall increased fungal load in IBD during disease flare, compared to healthy individuals (50, 51). IBD fecal samples generally display an increased Basidiomycota:Ascomycota ratio, increased *C. albicans* species, and decreased *S. cerevisiae* species, although discrepancies exist between studies (50, 51). Below we discuss the key microbial species identified to be abundant in healthy sections of the colon and the changes reflected in the IBD colon (**Figure 3**).

### Cecum

The cecum absorbs large volumes of water and electrolytes, and the microbes present here typically ferment carbohydrates (52). Studies of the healthy luminal microbiota of the cecum show that it is home to prevalent bacteria from the phylum Actinomycetota (formerly Actinobacteria), Bacteroidota (formerly Bacteroidetes), Bacillota (formerly Firmicutes), and Pseudomonadota (formerly Proteobacteria) (35, 53). Bacteria from the family *Baceteroidaceae*, *Rhodocyclaceae, Pasteurellaceae, Aeromonodaceae, Carnobacteriaceae*, *Prevotellaceae, Flavobacteriaceae, Enterococcaceae, Erythrobacteraceae, Sphingomonadaceae*, and *Alcaligenaceae* have been identified in healthy luminal cecum samples (35, 53–55). At the genera level, bacteria such as *Parabacteroides, Shigella, Dorea, Coprococcus, Blautia, Bacteroides, Alistipes, Lactobacillus*, *Bifidobacterium, Fusobacteria, Lachnospira, Enterococcus, Faecalibacterium, Roseburia*, *Escherichia, Prevotella*, and *Chryseobacterium*, along with a smaller populations of *Eubacterium, Clostridium*, and *Ruminococcus* were identified in the cecum (35, 53–55). Many of these microbes play a key role in the fermentation of non-digestible carbohydrates (resistant-starch and fiber) (21, 54). A preprint article looking into the mycobiome, using eukaryotic rRNA operon internal transcribed spacer-2 sequencing (ITS), in the colon of non-IBD individuals identified cecal fungal species, including members of the *Malasseziale* order, along with species *Malassezia restricta, Malassezia (ASV13), Cladosporiaceae (ASV16), Ramularia (ASV35), Penicillium paneum, Sporobolomyces johnsonii, C. albicans*, and *Cyberlindnera jadinii*, with a lower abundance of *C. albicans* in the cecum and ASC compared to other sections of the large intestine noted (56). Another study, also using ITS sequencing detected *Malassezia, Candida*, and, *Cladosporium* and found a higher abundance of the genera *Pichia*, *Fusarium*, and *Galactomyces*, compared to individuals with IBD (57). No studies of the cecal virome (eukaryotic viruses or bacteriophages) have been published in humans to date to the best of our knowledge.

Compared to the cecal mucosa of healthy controls, IBD patients have a decrease in bacteria from the Bacteroidota (formerly Bacteroidetes) phyla and an increase in Bacillota (formerly Firmicutes) (35). The Actinomycetota (formerly Actinobacteria), Proteobacteria, and Verrucomicrobiota phylum were found in IBD patient samples and supposedly not in healthy controls (35). A study looking at the cecal bacterial community in the mucosa of Chinese IBD patients found a higher abundance of the families *Rhodocyclaceae, Pasteurellaceae, Aeromonodaceae, Carnobacteriaceae*, *Flavobacteriaceae, Enterococcaceae, Erythrobacteraceae, Sphingomonadaceae*, and *Alcaligenaceae*, and a lower abundance of *Prevotellaceae* in CD patients; at the genus level *Prevotella, Coprococcus*, and *Blautia*, were decreased and *Chryseobacterium* and *Enterococcus* were increased in CD, compared to healthy controls (55). Another study found a decrease in the genera *Alistipes, Bacteroides, Dorea*, and *Parabacteroides*, and an increase in *Roseburia*, *Escherichia, Shigella*, and, interestingly, *Blautia* in IBD mucosal samples (35). In CD samples there was a decrease in *Coprococcus* and *Faecalibacterium* compared to healthy controls, but these were increased in UC samples (35). Other bacterial genera found the cecal mucosa of IBD patients have been identified including *Haemophilus, Klebsiella, Pseudomonas, Sporacetigenium*, *Akkermansia*, and *Holdemania* in UC samples, compared to healthy controls (35). In comparison to the mycobiome of healthy controls, the genus *Malassezia* is the predominant fungi identified in the mucosal samples of patients with CD, along with *Candida* and *Cladosporium* (57, 58). In addition, there is also a reduced population of *Pichia*, *Fusarium*, and *Galactomyces* in CD compared to healthy individuals (57). Interestingly, the cecum is also the site where the appendix, a thin tube-like independent extension, attaches to the intestinal tract (59). The appendix is thought to serve as a reservoir for beneficial microbes in healthy individuals (60) or possibly pathobiont microbiota in IBD patients (59). Therefore, as the cecum is in closest proximity to the appendix, the microbiota may be significantly influenced by the appendiceal microbiota.

### Ascending colon

Water and any remaining indigestible materials are further absorbed by the ASC, which solidifies food particles to form stool (61). ASC biopsies revealed the presence of bacteria from the phyla Thermotoga (formerly Thermotogae), Actinomycetota (formerly Actinobacteria), Bacillota (formerly Firmicutes), Pseudomonadota (formerly Proteobacteria), Bacteroidota (formerly Bacteroidetes), Mycoplasmatota (formerly Tenericutes), Cyanobacteria, Synergistota, Verrucomicrobiota, Deinococcus-Thermus, Aquificota, Lentisphaerota, Nitrospirota, Spirochaetota and Chloroflexota, along with the class Chlamydiae and Chlorobia, and the genera *Planctomycetes* and SRB (*Desulfovibrio*) (62, 63). A study by Chindi et al. sampled mucosal brushings from the ASC of male volunteers for the analysis of mucosa-associated microbiota using 16S sequencing (64). They found that the family level in the healthy ASC included bacteria *Bacteroidaceae, Bifidobacteriaceae*, and *Lachnospiraceae* which play an essential role in non-digestible carbohydrate fermentation and production of SCFAs (64). Mucosal samples of healthy individuals were also predominated by *Pseudomonadaceae*, *Xenococcaceae, Methylocytaceae, Bacillaceae*, and *Commonadaceae* families and the genera *Brevundimonas*, *Actinobacillus*, *Anaerostipes*, *Actinomyces, Peptostreptococcus, Parabacteroides, Pseudoxanthomonas, Eiknella, Streptococcus*, and *Roseburia* (37). A study looking at the mycobiome in the ASC mucosa of non-IBD individuals (preprint) found species from the order Malasseziales, and the genus *Phaeococcomyces*, along with species such as *M. restricta, Fungi (ASV09)*, *Fungi (ASV06), P. paneum, Yamadazyma mexicana*, *C. tropicalis*, *C. albicans*, and *C. jadinii* (56). Currently, there are no published studies of the ASC virome.

In the ASC of CD patients, there is an increase in pathobiont bacteria at the family level, including *Methylocystaceae* and *Comamonadaceae*, along with the genera *Actinomyces*, *Peptostreptococcus*, *Parabacteroides*, with a lesser increase in the family *Bacillaceae*, along with the genus *Pseudoxanthomonas*, *Eikenella*, and *Streptococcus* (37). Crypt mucosal biopsies have also shown an increase in SRB (*Desulfovibrio*) in patients with UC compared to healthy individuals (63). *Desulfovibrio* are typically considered resident commensals in the microbiota of healthy individuals however, they can transition into opportunistic pathobionts and increase in abundance within a dysbiotic microenvironment, such as UC (65). However, no studies have been performed on the ASC mycobiome or virome (eukaryotic viruses or bacteriophages) in IBD patients.

### Transverse colon

In addition to the absorption of water and nutrients, the main function of the transverse colon is sodium absorption (66). A study of mucosal biopsies from healthy Swedish volunteers displayed a high abundance of the classes Clostridia, Bacteroidia, Erysipelotrichia, along with the species *Bacteroides thetaiotaomicron*, *Bacteroides faecalis*, *Bacteroides uniformis*, and *Bacteroides caccae* in healthy individuals (67). In contrast, a smaller abundance of the classes Verrucomicrobiae and Coriobacteriia, and the genera *Desulfovibrio* and *Bacteroides* were also found in the healthy transverse colon (37, 67). In addition, carotenoid biosynthesis, which displays a protective role in the gut by regulating the intestinal immune responses, was found to be enhanced due to the presence of bacterial species *Bacteroides vulgatus*, *Akkermansia muciniphila*, *F. prausnitzii*, and *Parabacteroides distasonis* (67). Healthy mucosal biopsies display a high prevalence of other bacterial genera including *Clostridium, Enterococcus, Propionibacterium, Veillonella, Corynebacterium*, *Enterobacter, Klebsiella, Lactobacillus, Proteus*, and the species *E. coli* (68). A study by Kourkoumpetis et al. (preprint), mentioned earlier, also looked at the mycobiome community within the transverse colon of non-IBD individuals and found species from the order Malasseziales and species such as *Malassezia restricta, C. albicans, Malassezia (ASV13), Alternaria (ASV14), Bolbitius demangei, Saccharomycetales (ASV38)*, and *Fungi (ASV09)* (56). Another study also found the genera *Malassezia* in a healthy British cohort (69). There are no reports about the virome (eukaryotic viruses or bacteriophages) of healthy individuals in the transverse colon.

The *Bacteroides* genera are known to produce enzymes involved in tryptophan (Trp) metabolism which is reduced in IBD (67, 70). This suggests a potential depletion of *Bacteroides* in IBD patients (71). However, one species, *Bacteroides thetaiotaomicron* was found to be increased in mucosal transverse colon biopsies from CD patients (69). As seen in other segments of the colon, there is an increase in sulfur reducing bacteria (SRB; *e.g.*, *Desulfovibrio*) in transverse colon mucosal biopsies from patients with UC, compared to healthy controls (37). Another study showed an increase in the *Malassezia* genus in CD mucosa of British and Dutch cohorts compared to healthy controls (69). However, studies on the transverse colon bacteriome and mycobiome remain limited in IBD and there are no studies that have investigated the virome (eukaryotic viruses or bacteriophages) in the transverse colon of humans to date.

### Descending colon

The descending colon serves as a conduit which holds feces until it is discharged into the rectum (61). In healthy individuals, the descending colon is thought to be dominated by beneficial microbes such as the *Lachnospiraceae* family members, which are essential for protein fermentation (72). The healthy descending colon contains the phyla Bacillota (formerly Firmicutes), Pseudomonadota (formerly Proteobacteria), Bacteroidota (formerly Bacteroidetes), Actinomycetota (formerly Actinobacteria), Mycoplasmatota (formerly Tenericutes), Thermotogota (formerly Thermotogae), Synergistota, Deinococcus-Thermus, Chloroflexota, Lentisphaerota, Nitrospirota Aquificota, Verrucomicrobiota, Acidobacteria, Spirochaetota, and Cyanobacteria (62). Furthermore, members of the class Chlamydiae and Chlorobia have been identified in healthy descending colon biopsies (37, 62). At the family level, bacteria identified in the mucosa of the descending colon include *Micrococcaceae, Bacillaceae*, and *Commonadaceae*, along with lesser abundant families such as *Pseudomonadaceae* (37, 62).

At the genus level, *Actinomyces*, *Roseburia*, *Akkermansia* and *Streptococcus* are commonly identified in the healthy descending colon mucosa, while *Parabacteroides, Shigella, Brevundimonas and Ruminococcus* are found in lesser abundance (37). Furthermore, the genera *Planctomycetes*, SRB (*e.g.*, *Desulfovibrio*), *Fusobacteria*, *Eiknella, Peptostreptococcus, Marinilactibacillus, Proteus*, and *Pseudoxanthomonas* have been identified in biopsies from healthy descending colon (37, 62). The descending colon mycobiome of non-IBD individuals includes a high abundance of *M. restricta, C. albicans, Fungi (ASV09), P. paneum, Cladosporiaceae (ASV16), Ascomycota (ASV01)* and, to a smaller extent, *Ramularia (SV10)* and *Malasseziales* sp (56). No studies have been published characterizing the descending colon virome (eukaryotic viruses or bacteriophages) in healthy individuals to date.

In IBD patients, there is an increase in bacteria from the family *Bacillaceae* along with genera SRB (*e.g.*, *Desulfovibrio*), *Eikenella*, *Streptococcus*, *Peptostreptococcus*, *Marinilactibacillus*, *Proteus*, *Parabacteroides*, and *Ralstonia* in CD patients as evidenced by sequencing of mucosal samples (37, 63). Similarly, there is also a slightly increased abundance of the family *Comamonadaceae* as well as the genera *Actinomyces*, *Fusobacteria*, and *Pseudoxanthomonas* (63). Again, to the best of our knowledge, no studies currently exist on the virome (eukaryotic viruses or bacteriophages) and mycobiome of the descending colon in IBD patients (73).

### Sigmoid colon

The sigmoid colon is responsible for the transfer of stool into the rectum (61). Bacteria identified in healthy sigmoid colon biopsies were from the phyla Bacillota (formerly Firmicutes), Pseudomonadota (formerly Proteobacteria), Bacteroidota (formerly Bacteroidetes), Actinomycetota (formerly Actinobacteria), Mycoplasmatota (formerly Tenericutes), Thermotogorta (formerly Thermotogae) Synergistetota, Deinococcus-Thermus, Chloroflexota, Lentisphaerota, Nitrospirota, Aquificota, Acidobacteriota, Spirochaetota, Cyanobacteria, Verrucomicrobiota and Fusobacteriota, along with the class Chlorobia, and the genera SRB (e.g., *Desulfovibrio*) (62, 63). The sigmoid colon has a greater abundance of the genus *Bacteroides* compared to other sections of the colon (48, 74, 75). Other genera including *Veillonella, Clostridium, Corynebacterium, Sutterella*, *Lactobacillus, Klebsiella, Peptococcus* and *Enterobacter* were also found in high abundance in the mucosal analysis of non-IBD patients, along with other bacteria of smaller abundance such as *Enterococcus* and *Fusobacterium* (74–76). At the species level, high abundance of *E. coli, B. fragilis, F. prausnitzi*, *Haemophilus parainfluenzae*, and *Prevotella copri* were uncovered in the mucosal analysis of non-IBD patients (74–76). At the species level, *Methanobrevibacter arboriphilus, Methanobrevibacter smithii*, and *Methanosphaera stadtmanae* are found in high abundance in healthy individuals (77). In examining the healthy sigmoid mycobiome, *Candida*, *Pichia*, *Fusarium*, *Galactomyces, Malassezia*, and *Cladosporium* have been identified in the sigmoid colon mucosa (57). Again, no information could be found on the virome (eukaryotic viruses or bacteriophages) of the sigmoid colon in healthy individuals.

In IBD patients, there is a decrease in microbiota α-diversity [within each biopsy sample; Shannon index (4.25 vs 3.45) and Chao1 index (156.29 vs 98.67)] and a clear separation based on β-diversity analysis [between the biopsy samples; weighted and unweighted UniFrac with a PERMANOVA test (p = 0.001 for both)] in sigmoid colon mucosal biopsies, compared to healthy controls (76). Specifically, the inflamed mucosa in IBD patients was found to have a decrease in Bacteroidota (formerly Bacteroidetes) and Bacillota *(*formerly Firmicutes) and an increase in Pseudomonadota phyla, compared to healthy controls (76). When comparing IBD inflamed mucosa in flare to IBD patients not in flare, there was a decrease in *Ruminococcaceae*, *Lachnospiraceae*, and *Paraprevotellaceae* families, along with a decreased in genera *Bacteroides* and *Sutterella*, and species *B. fragilis, F. prausnitzii, H. parainfluenzae*, and *P. copri* (76). There is also an increase in the genera SRB (e.g., *Desulfovibrio*) in IBD biopsies (63). A study by Limon et al., analyzed the mucosal mycobiome and found that fungi belonging to the genera *Malassezia* and *Cladosporium* are found in higher abundance in patients with CD, compared to healthy controls (57). However, *Pichia*, *Fusarium*, and *Galactomyces* were found to be decreased in CD, compared to non-IBD along with a slight decrease in *Candida* as well (57). No studies have been conducted on the virome (eukaryotic viruses or bacteriophages) in the sigmoid colon to date.

### Rectum

The main role of the rectum is to store feces until it is expelled by defecation (8). Healthy rectal biopsies and swabs contain phyla Fusobacteriota, Bacillota (formerly Firmicutes), Pseudomonadota (formerly Proteobacteria), Bacteroidota (formerly Bacteroidetes), Actinomycetota (formerly Actinobacteria), Mycoplasmatota (formerly Tenericutes), Thermotogota (formerly Thermotogae) Synergistota, Deinococcus-Thermus, Chlorflexota, Lentisphaerota, Nitrospirota, Aquificota, Acidobacteriota, Spirochaetota, and Cyanobacteria (35, 62). At the class level, the rectum contains bacteria from Cholorobia (62, 63). The rectal mucosa of healthy individuals displays a predominance of families *Frankiaceae* and *Actinomycineae*, along with the presence of *Pseudomonadaceae, Spingomonadaceae, Lactobacillaceae*, and *Micrococcaceae* (35, 37, 62). Many bacterial genera have been identified in the rectal mucosa including *Dyadobacter, Curvibacter, Melissococcus, Variovorax, Larkinella*, *Actinomyces, Peptostrepococcus, Streptococcus, Marinobacter, Actinobacillus, Brevundimonas, Roseburia, Ruminococcus, Lachnospira, Lactobacillus, Allobaculum, Planctomyces, Novosphingobium, Methyloverstatilis, Skermenlla, Alistipes*, *Bacteroides*, *Blastia*, *Coprococcus*, *Dorea*, *Shigella*, *Oscillibacter*, *Parabacteroides*, *Pseudomonas*, *Subdigranulum, Desulfovibrio, Escherichia*, and *Faecalibacterium* (35, 37, 60, 62, 63, 67). Species specifically identified in the healthy rectal mucosa include *B. vulgatus, B. dorei*, and *B. nordii* (60, 67). The mycobiome of healthy individuals is thought to include a high abundance of *M. restricta, Malasseziales sp, C. albicans, Trichosporon* (*ASV3*4), and *Cladosporiaceae* (*ASV16*) and to a smaller extent *Yamadazyma mexicana, Sporobolomyces johnsonii*, and *Alternaria* (*ASV14*) (Preprint data) (56). The healthy rectal mucosa includes eukaryotic viruses and bacteriophages such as *Coccolithovirus*, *Minivirus*, *Orthopoxvirus, Phix174microvirus, P1virus, T4virus, P22virus*, *Orthopneumovirus*, *Lambdavirus*, and *Caudovirales* (78). In contrast, in the IBD there is an increase in the phyla Pseudomonadota (formerly Proteobacteria) and Actinomycetota (formerly Actinobacteria) and a decrease in Bacteroidota (formerly Bacteroidetes), Bacillota (formerly Firmicutes), and Fusobacteriota in the rectal biopsies compared to healthy controls (35). At the genus level, *Pseudomonas* was found to be highest in the rectum, compared to the ileum, cecum, and mid-colon in CD and UC patients (35). Compared to healthy rectal samples, there was an increase in the genera *Blautia*, *Shigella*, and *Escherichia* in CD and UC patient samples, *Bacteroides* in CD patient samples only (35), and *Desulfovibrio* in UC biopsies (63). The genera *Akkermansia* and *Sporacetigenium* were only found in UC rectal biopsies (35). Furthermore, there was a decrease in *Coprococcus*, *Faecalibacterium*, *Parabacteroides*, and *Pseudomonas* in IBD (35). Further, swab cultures from IBD patients confirmed the presence of fungal species such as *C. albicans, Candida* sp. non*-albicans, C. lusitaniae*, and *Candida kefyr* (79). Another study using enrichment of virus-like particles of Chinese individuals showed that patients with UC have an increase in the abundance of bacteriopahges and eurkaryotic viruses from the genus *Phix174microvirus, P1virus, Lambdavirus, T4virus, P22virus*, and *Orthopneumovirus* in their rectum but a decrease in mucosa *Caudovirales* phage diversity and richness compared with healthy controls (78).

## The strengths and weaknesses of commonly utilized methods of investigating gut microbiota

Currently, different techniques, ranging from traditional culturing methods to the most recent advanced metagenomic sequencing or next-generation sequencing (NGS) technologies, have been used to examine the microbiome in health and disease (80). However, much remains to be uncovered for a variety of reasons. Firstly, sampling issues not only cause difficulty in obtaining mucosal and luminal microbiota from select regions of the intestinal tract, but differences in collection and sample processing can also lead to variable results (3). Heterogeneity of the microbiome between and within patient’s also results in variable findings with most studies ignoring these factors when publishing their results (*e.g.*, time of day, age, sex, stress, diet, host factors, and environmental factors) (3). The vast majority of studies publishing microbiota data have a higher proportion of Caucasian male individuals while ignoring most other factors including diet, which is largely why there is such discrepancy in the literature when describing what a healthy microbiome is. This includes the preparation protocols that patients undergo prior to colonoscopy and endoscopy, which can have significant effects on the microbiota profiles (4). In addition, the vast majority are obligate anaerobes, which poses a challenge during specimen collection, transport, and storage (81).

16S rRNA sequencing, 18S rRNA sequencing, whole genome shotgun metagenomics, and internal transcribed spacer (ITS)-next generation sequencing (NGS)-based amplicon sequencing have been utilized to explore uncultivated gut microbial communities (82). Many research studies have relied on 16S rRNA amplicon sequencing only, which, while more affordable and accessible, offers little to no functional information (46). While many studies claim to have examined the “microbiota” using this technique, it identifies only 16S ribosome containing bacteria and limited fungi, entirely ignoring the gut virome (46). As such, while the gut contains an abundance of viruses (primarily bacteriophages) and there is a well-recognized role of bacteriophages, eukaryotic viruses, and viral stage (*i.e.*, lytic or lysogenic) in UC (Caudovirales class and families *Virgaviridae*, *Anelloviridae*, *Circoviridae*, *Picobirnaviridae*) and CD (Caudiovirales class and families *Siphoviridae*, *Myoviridae*, *Podoviridae*), the profile of the virome is not well defined for the specific sub-organs of the intestine (83–87). This is important as bacteriophages drive horizontal gene transfer between bacteria in the gut, and likely contribute to shaping the microbiome and immune responses in IBD (85, 88, 89). Meta-genomics and meta-transcriptomics on regionally gathered samples may provide novel information due to their ability to provide more in-depth sequencing and functional information (90). However, these techniques require higher sample biomass and are more prone to human DNA and transcript contamination (particularly in biopsy samples), which can typically be overcome through the removal of host DNA prior to sequencing (23, 90).

Meta-genomic analysis of stool samples is more common for analysis of gut microbiota compared to mucosal microbiota samples because stool allows for easier longitudinal investigations of study participants by non-invasive sample collection (91). Whereas mucosal intestinal brushings and washes are more difficult to obtain as longitudinal sample collection is reliant on follow-up endoscopy, which could require the participants to undergo non-essential surgical procedures (91). Furthermore, mucosal microbiota samples are collected following endoscopy preparation which has significant impacts on the microbiota composition; therefore, while mucosal samples can better reflect the precise microbiota of a defined intestinal location, the stool (luminal) microbiota reflects a more natural microbiota sample (92). Conversely, while much information about the human gut microbiota originates from analyses of stool samples, the stool microbiota is mixed with food residues and ingested microbial contamination, shedding intestinal mucosa, inhibitors that may impair PCR amplification/NGS procedures, and passing microbes (93). While the stool microbiota is easily accessible, it does not reflect the microbiota at the region-specific sites of the digestive tract, however it does represent the unique luminal microbiota community (94). Mucosa-associated communities are sampled either through mucosal washes/brushings or within biopsies (95, 96). Biopsy samples collected during endoscopy represent a mix of loose and strongly adherent mucosal layers (97). These samples may not fully represent the overall mucosa-associated microbiota, especially in patchy diseases like CD. Biopsy collection is also invasive and may contain high proportions of human DNA, which can interfere with microbial DNA analysis, limiting these samples to use of 16s rRNA methods primarily (98). Researchers have explored alternative methods for sampling low microbial biomass in the GI tract. One proposed approach involves using intestinal “lavage” samples or gut washes/brushings, which include fluid remaining in the bowel after bowel preparation (99). These gut wash samples contain a mix of luminal and loosely adherent mucosal communities. Gut washes are collected by flushing the mucosal surface with sterile saline and aspirating the resulting mixture of mucus, allowing for sampling of both the loose mucus layer interface (MLI) and the adherent mucosal layer (98, 100). MLI sampling has shown promise in providing sufficient material for multi-omic experiments and identifying novel taxa relevant to IBD (98, 101, 102). However, as mentioned earlier, colonoscopy preparation is known to impact gut microbiota composition (92) and significant differences have been noted between mucosal microbiota, biopsy microbiota, and stool microbiota composition (96, 103, 104). Another source of concern is the need for consistency of sample handling, often at the discretion of the study participants, which kits (e.g., OMNIgene and BIOME-Preserve) attempt to help researchers overcome (93). Evaluation of the traditional stool collection method versus OMNIgene GUT kit revealed a significant influence on microbiota composition, although the reliability of these kits is not yet fully confirmed and confirmation should be performed by users prior to proceeding with study recruitment (93). Moreover, the overall outcome of microbial samples, such as the genetic composition of gut microbes, is influenced by collection and storage conditions (105). For example, the composition of Bacillota:Bacteroidota (formerly Firmicutes:Bacteroidetes) phyla in fecal samples is significantly affected by storage temperature (106). Traditional at-home stool collection requires patients to freeze stool, although there is no way to accurately record patient adherence to appropriate collection methods. Recent studies have demonstrated that this can possibly be overcome with OMNIgene GUT kit as it claims to keep samples safe for up to 60 days at room temperature (107). While much progress is being made among studies when it comes to sample collection, handling, and microbiota identification methods, there remains considerable divergence of opinion on the optimal scientific strategy for examining the microbiome and the sub-biomes (bacteriome, mycobiome, virome) (108, 109). Findings of investigations employing different approaches are much more inconsistent for mycobiome and virome than studies of the bacteriome, for example (51).

Lastly, while identification methods have provided vast amounts of information about the microbiota to date, microbiome exploration is further hampered by live-model flaws (110). For example, it is difficult to recapitulate the precise microenvironment of the gut for the live culture of microbiota communities (111). Researchers utilize variable culture conditions such as aerobic culture versus anaerobic culture, different culture media that do not entirely represent the gut microenvironment, and culture methods which lack mechanical microenvironment factors such as fluid flow, villi architecture, and peristalsis (111). Many microbes are difficult or arguably impossible to culture in a laboratory setting, and some select microbe species are well known to outcompete their community members, producing a culture unlike that of the sample source (112). As a result, simulating the entire activities of the human digestive system and real-time observations of interaction dynamics are difficult (113). While mouse models are the traditional animal of choice in many research studies, the pig shares clear microbiome similarities over other non-primate models in digestive tract anatomy, physiology, and immune response when compared to humans (114, 115). In addition, pigs and humans share more non-redundant genes in their microbiome than other model organisms, such as mice (116). While of course, humanized axenic mouse models present another opportunity to investigate the impacts of the gut microbiome in health and disease (117).

Currently, there is a lack of published literature regarding both the mycobiome and virome (50, 118). Initially recovering the fungal DNA is troubled by the thick cell wall (119). Further, sequencing technologies have not been well-adapted to identify species in the mycobiome, with different fungal extraction methods from fecal samples potentially driving the variation in results between studies (119). As well, the ITS, which are the preferred method for identifying fungi, vary in length between species and quality reference databases are lacking, leading to a lack of confidence in identification (119). The gut virome is a relatively new field of study and most of the studies to date have been limited to fecal samples (84, 120). Further, there are limited complete viral genome sequences, including sequences for bacteriophages, compared to bacterial genomes, troubled by viruses lacking an evolutionary conserved marker (*e.g.*, 16S rRNA), leading to a significant volume of unidentified species during bioinformatics analysis of sequenced datasets (82, 84, 86, 120, 121).

## Common considerations for microbiota research moving forward

The growing need to understand the regional composition of gut microbial communities as well as their significance to health and disease is an important step to enhance our understanding of the precise role of the gut microbiome in these settings. Recognizing the variability in microbiota communities housed in the various sub-organs of the intestinal tract, described in this review, future research should emphasize sampling different segments of the intestine and greater care should be taken with regard to communicating the precise location that samples (such as biopsy, gut brushings and gut washes) were collected in published manuscripts. The growing need to bridge the gap in healthcare requires collaboration among medical laboratory personnel, clinicians, and researchers studying the gut microbiome. However, the invasive nature of sampling techniques poses challenges in recruiting participants and obtaining a large variety of clinical samples from each participant (122). As a result, low sample sizes can impact the statistical power and generalizability of research findings (123). To overcome these obstacles, careful research planning, collaboration with experts, and clear communication with participants are essential (124).

In conclusion, this review highlights some of the key differences identified to date in the communities of microbes that take residence in the various segments of the intestinal tract in both healthy individuals and patients living with IBD. With rising incidence rates of IBD globally and significant recognition of the role of the microbiome in IBD, it is more imperative than ever that we improve our understanding of the microbiome in health and disease through improved sample collection, processing, research techniques, and reporting in peer-reviewed manuscripts (125).

## Author contributions

HA and SL conceived, developed, and coordinated the project. SL, AV, MB-J, HO, and HA drafted the manuscript. AV, HO, SL, and HA were responsible for the figure preparation. All authors contributed to the article and approved the submitted version.
